# We Were All Once Young: Reducing Hostile Ageism From Younger Adults' Perspective

**DOI:** 10.3389/fpsyg.2022.793373

**Published:** 2022-03-24

**Authors:** Zizhuo Chen, Xin Zhang

**Affiliations:** ^1^School of Psychological and Cognitive Sciences, Peking University, Beijing, China; ^2^Beijing Key Laboratory of Behavior and Mental Health, Peking University, Beijing, China

**Keywords:** hostile ageism, interventions, intergenerational relations, stereotype content model, dictator game

## Abstract

The worldwide spreading pandemic, COVID-19, has caused hostile ageism toward older adults. We adopted a new intervention to reduce such hostile ageism. “Imagine that they were Young” referred to the imagination of what an older adult might look like, think, and behave when they were once young, which was a reversed but refined intervention of the widely-used method of “Imagine that you were old.” In the present study, intergenerational tension was primed, and then 205 younger adults in China aged 18–37 were randomly assigned to 3 different conditions (“Imagine that they were Young,” “Imagine that you were old,” and control condition), asking them to imagine (and then write down) once older adults were young, or a future aging self, or read an unrelated essay respectively as experimental manipulations. Then they should distribute medical funds worthy of Chinese 1 million to two patients with COVID-19 of 25 and 85 years old indicating their attitudes toward older adults (or hostile ageism). Finally, we measured their general attitudes and stereotypes toward older adults. Results verified the effectiveness of both interventions, such that younger adults who took either intervention distributed more medical funds and showed more positive aging attitudes toward older adults than those in the control group. Moreover, “Imagine that they were Young” was tested to be even more effective than “Imagine that you were old.” A series of relative mediation models revealed that the stereotype of warmth mediated the effect for both interventions on decreased hostile ageism behaviors, compared with the control condition. While “Imagine that they were Young” could additionally reduce hostile ageism through a higher level of “including the older adults in their self-group (the young).” This new intervention might be a good alternative to eliminate hostile ageism.

## 1. Introduction

Ageism, a form of discrimination against older adults, was first conceptualized by Butler (1969) and is taken seriously nowadays (Ayalon and Tesch-Römer, 2018). Ageism will not only aggress upon the social life and rights of older adults but could also cause negative outcomes in cognitive performance (Levy, 1996), physical health (Levy et al., 2006, 2009), and even longevity (Levy et al., 2002). However, compared with sexism and racism, there is much less attention on ageism in our society and research (North and Fiske, 2012).

The worldwide spreading pandemic, COVID-19, has directly brought ageism to our society (Jimenez-otomayor et al., 2020; Choi and Yang, 2021). For example, Xiang et al. (2021) analyzed the comments concerned older adults on Twitter during COVID-19 and found over 10% of tweets showed ageism. Ng et al. (2021) also found increasing ageism narratives during COVID-19 across 20 countries. It was also reported that older adults' interests may be at the risk of being ignored or even sacrificed to mitigate the pandemic's negative outcome on younger generations in some countries (Ayalon et al., 2021; Barrett et al., 2021; Lichtenstein, 2021), which was also named “Senicide” by scholars (Xiang et al., 2021). All those expressions of ageism would violate older adults on their legitimate rights. (Chang et al., 2020; Fraser et al., 2020). Hence, it deserves our attention on how to intervene and reduce such ageism under COVID-19 contexts.

### 1.1. The Hostile Form of Ageism and the Intervention

Older adults may have experienced different forms of ageism during the pandemic which would exert various influences on them. Cary et al. (2016) divided ageism into two forms, namely, benevolent and hostile ageism. Benevolent ageism refers to the overwhelmed empathy and pity concerning older adults, which often leads to unwanted help (for example, providing extra but unnecessary medical treatment to older adults) and then results in decreased self-esteem, as well as motivation and confidence loss of older adults (Baltes and Wahl, 1996; Hess, 2006; Hehman and Bugental, 2015). Unlike the benevolent one, hostile ageism always refers to more aggressive and drastic attitudes and behaviors as we have witnessed in the case of COVID-19 (Ayalon et al., 2021). Undoubtedly, hostile ageism would put older adults at more urgent risk and call for a priority of intervention.

Indeed, hostile ageism reflects intergenerational tension in our society. For instance, younger adults may regard (or consider) older adults as a burden to society (Cohn-Schwartz and Ayalon, 2021), exert parasitic stigmatization on older adults (Cottrell and Neuberg, 2005), or use epithets like “boomer remover” on the internet during the pandemic (Lichtenstein, 2021). As intergroup threat theory (Riek et al., 2006) emphasized, younger adults would incline to regard older adults as a threat to their self-interest due to intergeneration tension (Ayalon, 2019), from which the hostile ageism originated.

Hence, from such an intergenerational perspective, it is of practical importance to develop interventions to alleviate the tension and in turn reduce hostile ageism. One prevailing intervention invites younger adults to imagine their future aging self (Packer and Chasteen, 2006), or look at the processed photos of future aging self through digital applications (Rittenour and Cohen, 2016), all of which were tested effectively on ameliorating intergenerational relationships. This “Imagine that you were old” intervention reminds younger adults of the destined aging fact (“All we will get old”) and consequently drives them to include the older adults into their in-group and then bring relief to intergenerational tension. As a result, such imagination of the aging self could lead to a more positive stereotype of aging (Packer and Chasteen, 2006; Rittenour and Cohen, 2016) and then promoted prosocial behaviors among younger adults through more pity and fewer envy feelings toward older adults (Rittenour and Cohen, 2016) as predicted by BIAS map [Behaviors from Intergroup Affect and Stereotypes, Cuddy et al. (2007)]. That is to say, “Imagine that you were old” could reduce hostile ageism through an increasing positivity of the aging stereotype. However, the lack of experience and knowledge on aging among younger adults would certainly cause restricted advantages of this intervention.

### 1.2. From “Imagine That You Were Old” to “Imagine That They Were Young”

Although evidence has revealed the effectiveness of the “imagine that you were old” intervention (Packer and Chasteen, 2006), one caveat should also be noticed that existing stereotypes may affect the imagination about the future aging self in a negative sense. It is suspected that such imagination of the aging self may simultaneously evoke the fear about aging or death (especially in COVID-19, with a higher probability of getting infected or death for older adults), and then inversely prompt the younger adults to keep their distance from their fragile older counterparts. As Packer and Chasteen (2006) have found, younger adults who held steady identity about their own age group would instead perceive older adults more negatively after imagining an aging self.

Therefore, we developed a new intervention, namely “Imagine that they were young,” to overcome these challenges. “Imagine that they were young” induced younger adults to imagine what the older adults would look like, behave and think when they (older adults) were once as young as themselves (young participants). Compared with “Imagine that you were old,” imagining a rejuvenated older adult involved less about aging stereotypes and younger adults were much more familiar with younger than older people's lives. Such intervention is in line with the perspective of “Inclusion of Outgroups in the Self” (IOS, Aron et al., 1992; Cadieux et al., 2019), which was defined as to what extent ingroup members perceive the outgroup ones are similar to them and include them into self-group. The view is surely related to how we interact with and perceive older adults. For example, Cadieux et al. (2019) found that IOS mediated the positive effect between the quality of intergenerational contact and positive attitudes toward older adults. From this IOS view, “Imagine that they were young” could directly fulfill the aim of putting older adults (out-group members) into younger adults' self-group by reminding them “they, older adults, were once members of your self-group.” In contrast, the “Imagine that you were old” urges the younger adults to include themselves into the “older adults,” which was indeed in direct contrast to IOS. Thus, we believe the “Imagine that they were young” intervention should be a more effective and practical way to reduce hostile ageism through an increase in the level of IOS among younger adults.

### 1.3. The Present Study

Current research took intergenerational relationships and IOS into consideration. Based on “imagine that you were old,” we raised “Imagine that they were young” as a more effective new strategy and we ought to investigate whether such “Imagine that they were young” would function well in reducing hostile ageism. We would further like to explore the potential mechanisms of the two interventions on the reduction of hostile ageism. In particular, we examined the mediation roles of aging stereotypes (i.e., warmth and competence) and the level of IOS.

In the present study, we measured not only general ageist attitudes with self-report scales but also using a hypothetical dictator game with contextual information available (i.e., participants were asked to distribute a certain number of resources under the COVID-19 pandemic) for two reasons. First of all, most previous studies only used self-report scales to represent explicit ageist attitudes which could induce severe social desirability effect, making participants more likely to conceal their ageist attitudes (instead of behaviors) toward older adults (Cherry et al., 2015). Therefore, we could minimize the influence of social desirability as such a hypothetical scenario could indeed reflect participants' implicit attitudes toward older adults. Moreover, sometimes, hostile ageism was rare but noticeable in our daily life (Marti et al., 2000), as it always occurred under very concrete social contexts (Ayalon and Tesch-Römer, 2018). In other words, as the finite(or scarce) resource perspective proposed (North and Fiske, 2012), the context of intergenerational competition on scarce public health goods during the pandemic (but not the general daily life) would motivate the younger adults to act hostilely against older adults. Hence, a dictator game with hypothetical scenarios would be more appropriate to capture such attitudes.

In summary, in the present study, we conducted one experiment to test and compare the effectiveness and possible mechanism across these two interventions (i.e., “Imagine that you were old” vs. “Imagine that they were young”) on hostile ageism by resource distribution tasks (North and Fiske, 2013) under COVID-19 pandemic. Specifically, we hypothesized that:

***Hypothesis 1***: Compared with the control condition, participants who took any one of these two interventions expressed less negative hostile ageism attitudes, behaviors, aging stereotypes, and a higher level of IOS;
***Hypothesis 2***: Compared with the “imagine that you were old” intervention, participants who took “Imagine that they were young” intervention expressed less negative hostile ageism attitudes, behaviors, aging stereotypes, and a higher level of IOS;

Beyond these two hypotheses, we were to test a series of relative mediation models in which stereotypes of warmth, competence, and level of IOS would play a mediation role respectively on the effect from either intervention to hostile ageism compared with the control condition. We expected to find convincing evidence to encourage a more specific explanation of how the two interventions worked and the differences between the mechanisms.

***Hypothesis 3***: Compared with the control condition, both two interventions could reduce hostile ageism through increased positivity of aging stereotypes (i.e., warmer and/or more competent).
***Hypothesis 4***: Compared with the control condition, the “Imagine that they were young” intervention could reduce hostile ageism through an increased level of IOS.

## 2. Method

### 2.1. Participants

Before data collection, the power analysis was conducted for sample size determination, and G*power software recommended 207 participants on conducting one-way ANOVA under 3 conditions (we set the effect size equal to 0.25, α error probability equal to 0.05, and test power equal to 0.90). Participants were recruited through an online platform named Credamo (https://www.credamo.com/home.html#/). The platform allowed us to display videos online and participants were restricted to those who could respond with computers rather than smartphones. After we had uploaded our experiment on this platform, 250 participants participated and finished our experiment. They were paid 8 RMB (equal to 1.3 USD) as a reward. Of the 250 participants, 45 responses (18% of total) over 40 years old, or who failed the attention check questions were discarded. A total of 205 responses aged 19 to 37 entered the final analysis. (Average age = 26.97, SD = 4.16, female = 62.4%).

### 2.2. Measures

***Hostile ageism behaviors***. Dictator games (Tversky and Kahneman, 1982) which means any decision made by participants would be accepted (as so-called dictators), could be used to reflect out-group discrimination behaviors (Fershtman and Gneezy, 2001). Thus, we designed a dictator game to simulate ageism decision-making under COVID-19 contexts as follow.

Participants were told to allocate a total of 1 million RMB as medical funds to two COVID-19 patients of different ages (a 25-year-old and another 85-year-old, with no mention of gender). Participants were also informed that the basic probability of cure was 25% for the young patient and 0 for the old, with every 10k medical funds allocated contributing to a 1% increase in the probability of cure. For younger patients, 750k medical funds would guarantee a 100% cure (and the remaining funds would only increase the probability of cure for the 85-year-old adult to 25%), while for older adults, only if they received the whole 1 million funds would guarantee a 100% cure (also only 25% probability of cure for the 25-year-old adult instead). Such a scenario creates a sense of resources scarcity, which would lead to intergenerational tension (as well as competition). Their decisions of allocation would certainly reflect their attitudes toward older (vs. younger) adults, or in other words, the (hostile) ageism.

***Ageism attitude***. We used the intergenerational tension ageism scale (North and Fiske, 2013) including three dimensions, such as consumption (on passive, shared-resource-consumption from older adults, e.g., “Doctors spend too much time treating sickly older people.”), succession (derived from expectations about enviable resources and societal positions possessed by older adults, e.g., “Most older people don't know when to make way for younger people.”), and identity (focusing more on symbolic resources like culture or lifestyle, e.g., “Generally older people shouldn't go clubbing.”) with 20 questions to measure explicit ageism attitudes toward older adults, using a 6-Likert Scale from “1-strongly disagree” to “6-strongly agree.” Participants rated higher on this scale (these three sub-scales) may hold more ageism attitude (of these three dimensions) toward older adults. Cronbach's Alpha was 0.801 for succession, 0.775 for identity, 0.706 for consumption dimension, and 0.877 overall.

**Figure 1:**
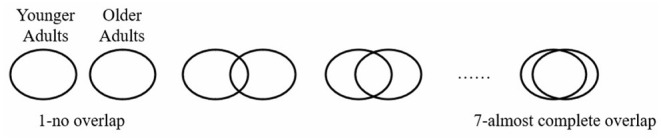
Measuring levels of Inclusion of Outgroups in the Self (IOS).

***Inclusion of outgroups in the Self***. We adopted the Venn diagrams test developed by Aron et al. (1992) to measure IOS. The scale consists of 7 different pairs of two circles representing the younger and older adults, respectively. The degree of overlap of the two circles represents to which degree participants perceived the similarity shared by the younger and older adults. They should choose one of these 7 different forms from “1-no overlap” (lowest IOS) to “7-almost complete overlap” (highest IOS) as Figure 1.

***Aging stereotype***. Participants had to rate a 9-Likert scale from “1-not at all” to “9-totally identical” on 10 adjectives describing older adults. These 10 adjectives were derived from the Stereotype Content Model (Fiske et al.,^2002^, Study 2), with 5 reflecting “warmth” (friendly, trustworthy, warm, well-intentioned, and sincere) and the other 5 reflecting “competence” (capable, efficient, intelligent, skillful, and confident). The higher score indicated more positive aging stereotypes. Cronbach's Alpha was 0.815 for “warmth” and 0.824 for the “competence” dimension in our study.

### 2.3. Procedure

After getting participants' formal consent, they were asked to read the “finite resource across age groups” passage to prime their hostile ageism against older adults (we conducted a pilot study to determine the effectiveness of such priming, and also a manipulation check for our study, refer to Supplement Materials for details). Then they were randomly assigned to the following three conditions.

***“Imagine that you were old” condition***. Participants watched a 3-min video [ImagineVideoclips (2019), for online resource] presented 101 strangers aged 0–100 years old, respectively, speaking out their age in order (from 0 to 100). After that, we presented an image including two men (one 26 years old and another 82) and two women (one 25 years old and another 78, this image was a screenshot from the previous video). Then younger participants have to imagine that when they were as old as the older people appeared in video and image. They were asked to write down what they would look like, what their characteristics would be, and what would happen in their life at that time (no less than 100 Chinese characters).

***Imagine that they were young condition***. Here, the participants watched the same video showing 101 strangers. The only difference was the inverse appearance order (from 100-year-old stranger to 0-year-old baby). After that, they looked the same image with 4 particular strangers. Then they have to imagine that when the older adults as what appeared in video and image were as young as the younger participants. They were asked to write down what the strangers would look like, what their characteristics would be, and what would happen in their life at that young (also no less than 100 Chinese characters).

***Control condition***. Participants in the control group read an article named “Sauropod, the Titan on our planet” derived mainly from Wikipedia and were rewrote by researchers. After reading that, participants should answer four questions about the main content of the passage.

Next, we measured their hostile ageism behaviors (allocating medical funds), level of IOS, aging stereotype, ageism attitude and recorded their age and gender in order. Finally, we thanked their participation and presented anti-ageism material for debriefing.

## 3. Results

### 3.1. Descriptive Statistics

In Table 1, we presented the demographics (i.e., age and gender), medical funds allocation, level of IOS, aging stereotypes (warmth and competence), and three types of ageism attitudes (succession, consumption, and identity) under two interventions and the control condition. There were no differences in gender composition (χ^2^ = 0.389, *p* = 0.823) and age (*F*_(2, 202)_ = 0.296, *p* = 0.744) across three conditions.

**Table 1:** Main dependent and independent variables across three conditions [M(*SD)*].

**Interventions**	**Imagine that you were old**	**Imagine that they were young**	**Control Condition**
*N*	69	59	77
Proportion of Female	63.77%	64.41%	59.74%
Age	27.2*(4.34)*	26.6*(3.50)*	27.1*(4.48)*
Medical funds to older adults (RMB Yuan)	485k*(153k)*	539k*(152k)*	441k*(177k)*
Level of IOS	4.49*(1.11)*	4.78*(1.13)*	4.3*(1.09)*
Competence	5.69*(1.25)*	5.74*(1.27)*	5.34*(1.25)*
Warmth	7.38*(0.92)*	7.43*(0.85)*	6.93*(1.02)*
Consumption	2.67*(0.68)*	2.69*(0.66)*	2.69*(0.74)*
Succession	3.28*(0.79)*	3.43*(0.76)*	3.50*(0.79)*
Identity	2.39*(0.78)*	2.48*(0.83)*	2.31*(0.90)*

*The italic values in parenthesis stand for Standard Deviations*.

### 3.2. Interventions on Hostile Ageism

**Figure 2:**
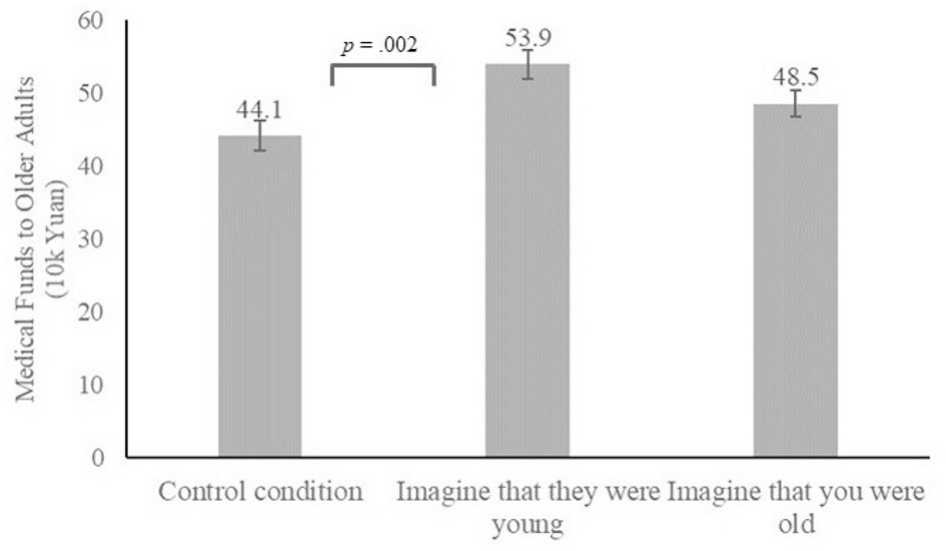
Hostile ageism behavior across three conditions. Error bars stand for the SEM.

***Hostile ageism behavior***. A one-way ANOVA with conditions (“Imagine that they were young,” “Imagine that you were old” and control condition) as a between-subject factor on medical funds allocated to older patients was conducted. Results of one-way ANOVA revealed significant differences across the three conditions (*F*_(2, 202)_ = 6.191, *p* = 0.002, partial η^2^ = 0.058) as Figure 2 on medical funds allocated to older patients. Bonferroni's *post-hoc* test showed that only the participants who “imagined that they were young” (*p* = 0.002) allocated more medical funds to older adults than those in the control condition. Instead, participants who “imagined that you were old” did not outperform those in the control condition (*p* = 0.292). However, no significant difference emerged between participants who took “Imagine that they were young” and “Imagine that you were old” intervention (*p* = 0.187).

***Ageism attitudes***. A 3(conditions, between-subject) *3(dimension of ageism attitude, within-subject) mixed model ANOVA showed only one significant main effect of dimensions on ageism attitudes (*F*_(2, 202)_ = 203.39, *p* < 0.001, partial η^2^= 0.502). The *post-hoc* test revealed that participants were more likely to perform ageism of succession than consumption (*p* < 0.001) or identity (*p* < 0.001), and perform ageism of consumption than identity (*p* < 0.001). However, no significant differences across conditions (*F*_(2, 202)_ = 0.32, *p* = 0.73, partial η^2^= 0.003) and interaction (*F*_(2, 202)_ = 1.95, *p* = 0.102, partial η^2^= 0.019) emerged.

***Aging stereotypes***. A 3(conditions: “Imagine that they were young,” “Imagine that you were old” and control condition, between-subject) *2(dimension of stereotypes: “warmth” and “competence,” within-subject) mixed-model ANOVA showed significant main effect for both factors but insignificant interaction(*F*_(2, 202)_ = 0.18, *p* = 0.84, partial η^2^ = 0.002). For stereotypes, participants were more likely to rate older adults as warmer rather than more competent (*F*_(1, 202)_ = 397.05, *p* < 0.001, partial η^2^ = 0.66). For different conditions (*F*_(2, 202)_ = 4.91, *p* = 0.008, partial η^2^= 0.046), *post-hoc* test further revealed that participants in both “imagined that they were young” (*p* = 0.019) and “imagined that you were old” (*p* = 0.033) conditions perceived older adults as more positive (i.e., higher ratings of warmth and competence) than those in the control condition.

***Level of IOS***. A one-way ANOVA with conditions (“Imagine that they were young,” “Imagine that you were old” and control condition) as a between-subject factor on the level of IOS was conducted. Results found significant differences across the three conditions (*F*_(2, 202)_ = 3.16, *p* = 0.045, partial η^2^ = 0.030). The *post-hoc* test revealed that only participants who took he “Imagine that they were young” intervention showed a higher level of IOS than those under the control group (*p* = 0.038). While participants who took the “Imagine that you were old” intervention showed no significant difference in the level of IOS compared with participants under either control (*p* = 0.88) or “Imagine that they were young” (*p* = 0.44) condition.

### 3.3. Pathways From Interventions to Hostile Ageism

Concerning the multi-categorical independent variables (3 conditions), three **relative mediation models** with different mediators (***M***) (i.e., the aging stereotype of warmth, the aging stereotype of competence, and level of IOS, respectively) were analyzed separately (Hayes and Preacher, 2013, 2014). We, additionally, conducted a single model with all these three mediators together in parallel. Detailed results of this model could be found in Supplementary Materials. Two dummy variables **D**_1_ (1 = “Imagine that they were young” condition, 0 = other conditions) and **D**_2_ (1 = “Imagine that you were old” condition, 0 = other conditions) were generated to represent different interventions. Medical funds allocated to older adults (***Y***) were used as the dependent variable, which reflected **non**-hostile ageism. For hostile ageism attitudes, no significant differences emerged across conditions as we found above. Therefore, we did not conduct the relative mediation analysis using hostile ageism attitudes as dependent variables. We then used the PROCESS macro developed by Hayes and Preacher (2013) in SPSS to test these relative mediation models.

First of all, for all three relative mediation models, relative total effects reached significance for variable **D**_1_ (i.e., “Imagine that they were young” condition, 95% Bootstrap CI = [4.34, 15.42], *p* < 0.001) and marginal significance for variable **D**_2_ (i.e., “Imagine that you were old” condition, 95% Bootstrap CI = [-0.82, 9.79], *p* = 0.097). Omnibus test of total effect of conditions on ageism behaviors showed significant (*F*_(2, 202)_ = 6.19, *p* = 0.003, partial η^2^= 0.058).

**Figure 3:**
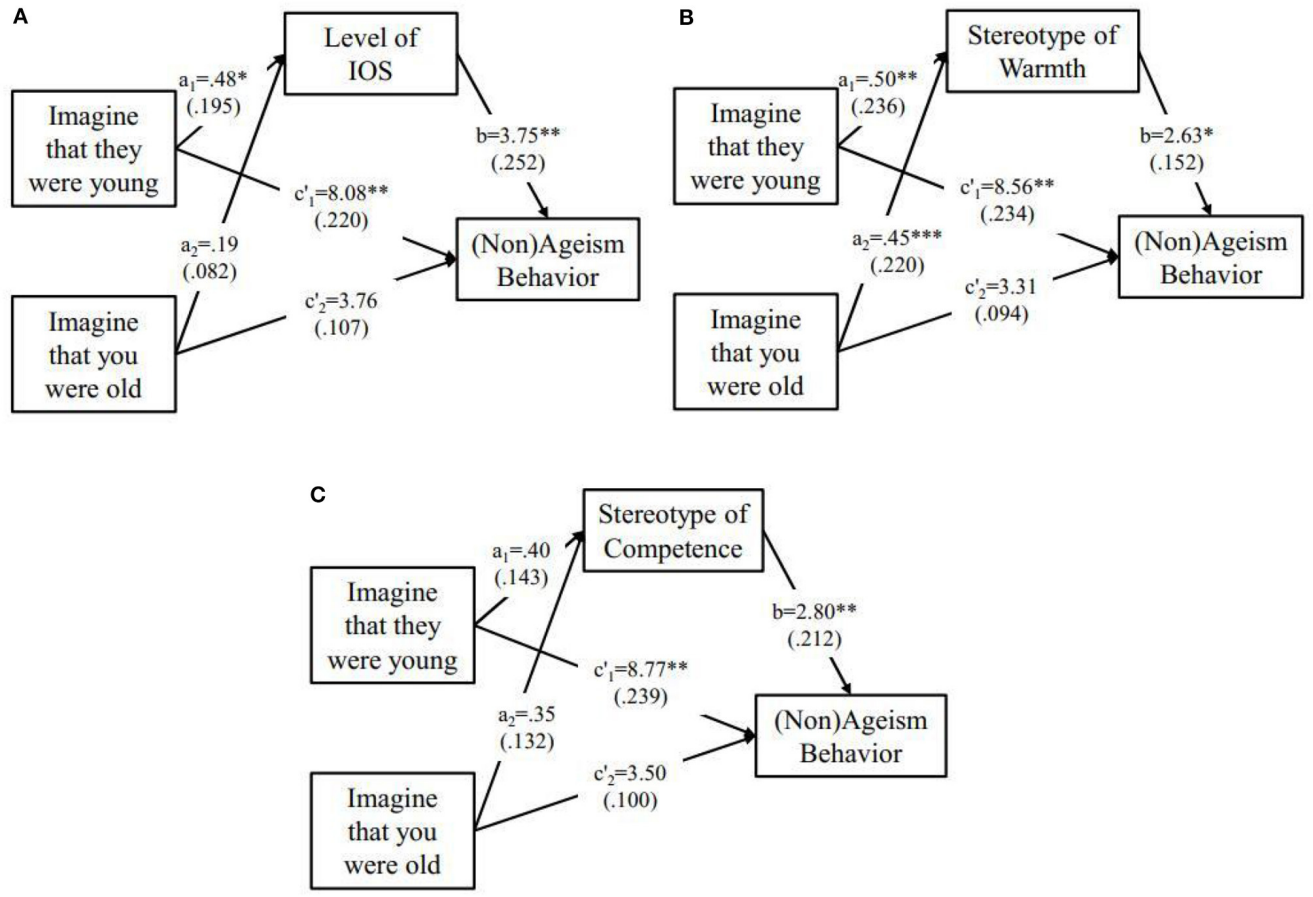
Relative mediation model of warmth **(A)**, level of IOS **(B)**, and competence **(C)** on effect of interventions (compared with control condition) to (Non)hostile ageism behaviors, for which was measured as medical funds allocated to **older adults**. Standardized path coefficients were presented in parentheses. *p < 0.05; **p < 0.01; ***p < 0.001.

As for the level of IOS, omnibus test of direct effect showed significant (*F*_(2, 201)_ = 4.28, *p* = 0.015, partial η^2^= 0.041). Only the indirect effect of variable **D**_1_ (95% Bootstrap CI = [0.30, 3.85]) but not **D**_2_ (95% Bootstrap CI = [0.30, 3.85]) reached significant, which indicated that “Imagine that they were young” condition could reduce hostile ageism (*b* = 3.75) by including older adults more (*a*_1_ = 0.48) into their self-group. (Please refer to Figure 3A).

As for stereotypes of warmth, omnibus test of direct effect showed significant (*F*_(2, 201)_ = 4.55, *p* = 0.012, partial η^2^ = 0.043). Significant relative indirect effects emerged for both variable **D**_1_ (95% Bootstrap CI = [0.20, 2.71]) and variable **D**_2_ (95% Bootstrap CI = [0.14, 2.52]). To be specific, participants who took either “Imagine that they were young” or “Imagine that you were old” intervention rated older adults as 0.50(*a*_1_) or 0.45(*a*_2_) higher on warmth, respectively, than the control condition, which lead to a significantly reduced hostile ageism (*b* = 2.63). (Please refer to Figure 3B).

As for the stereotypes of competence, omnibus test of direct effect showed significant (*F*_(2, 201)_ = 5.02, *p* = 0.008, partial η^2^= 0.048). However, no significant relative indirect effect emerged for either variable **D**_1_ (95% Bootstrap CI = [-.01, 0.16]) or variable **D**_2_ (95% Bootstrap CI = [-.01, 0.14]), which indicated stereotype of competence did not serve as mediator on reducing hostile ageism. (Please refer to Figure 3C).

## 4. Discussion

In the present study, we investigated whether and how “imagine that you were old” and the new intervention “Imagine that they were young” could reduce hostile ageism under the COVID-19 pandemic. Results of the current experiment partially confirmed our original Hypothesis 1, such that “Imagine that they were young” could function well as expected, while “Imagine that you were old” only showed a marginally significant effect in reducing ageism. Results also supported our second hypothesis that our new intervention of “Imagine that they were young” outperformed the “Imagine that you were old” intervention, in terms of their medical fund allocations. Moreover, a series of mediation models further explored possible mechanisms and illustrated that both interventions could lead participants to perceive older adults as warmer (one potential mediator) and resulted in lower hostile ageism behaviors as Hypothesis 3 predicted. In addition, as Hypothesis 4 expected, participants in the “Imagine that they were young” condition (but not in the “Imagine that you were old” condition), compared to those in the control condition, showed a significantly higher level of IOS (i.e., showed more preference to include older adults into their self-group,) as well, which also lead to reduced hostile ageism.

### 4.1. “Imagine That They Were Young,” a Better Choice?

Although both interventions could successfully reduce hostile ageism to some extent, our newly developed, “Imagine that they were young” intervention outperformed “imagine that you were old” in terms of effectiveness. From our mediation analysis, it is revealed that the perceived warmth of older adults could lead to reduced hostile ageism for both “Imagine that you were old” and “Imagine that they were young.” Indeed, as suggested by the BIAS map (Cuddy et al., 2007), holding stereotypes of warmth (as well as incompetence) made younger adults feel pity or sympathetic toward older adults, and then induced more prosocial behavior like what we have observed in the current experiment. Beyond the pathway of warmth shared by both interventions, the “Imagine that they were young” reduced hostile ageism additionally by including the older adults into younger participants' self-group as well (as indexed by enhanced IOS), which may be the reason why this new intervention worked better. As we proposed, such imagination reminded younger adults that their older counterparts were once members of their in-group, with the same life events (for example, one participant wrote “he was not so good at math and spent a lot of time on solving hard questions,”) looks, characteristics, or expectations about the future. Therefore, confronting older adults, younger participants would then take more account of all those similarities rather than chronological age *per se*.

Moreover, as expected, such inclusion (i.e., a higher level of IOS) could predict less hostile ageism. Cadieux et al. (2019) found that younger adults' positive contact experiences with older adults could reduce incompetence stereotypes through an increase in IOS, and such an increase in IOS could also lead to more positive attitudes toward older adults. Instead, “Imagine that you were old” that “Including myself (i.e., *younger adult*) eventually into an *out-group*” was in direct contrast to “Including an *older adult* into the *in-group*” by definition. Besides, as terror management theory (Becker, 1973) claimed, “Imagine that you were old” may indeed induce certain anxieties toward own aging and make young participants keep their distance from older adults (Martens et al., 2005). Furthermore, such “Imagine that you were old” could even evoke ageism in terms of self-esteem defense as suggested by social identity theory (Tajfel and Turner, 2004). Rittenour and Cohen (2016) found that younger participants who viewed a transformed image of the aging self were more likely to express negative aging attitudes and deny the authenticity of the aging-self simulation. In fact, there have been other studies failing to find the effectiveness of “imagine that you were old” [e.g., Packer and Chasteen (2006)]. Our data provided further support on why such “Imagine that you were old” might not work, from the perspective of IOS.

### 4.2. Ratings of Warmth but Not Competence as a Mediator

Although, warmth ratings were confirmed to be an important antecedent in affecting participants' hostile ageism. However, the stereotype of competence (particularly incompetence) failed to have any effect on reducing hostile ageism in our study, which is contrary to the BIAS map. For example, Lytle et al. (2020) found that younger adults who viewed older adults as incompetent showed more intention to help under COVID-19 circumstances. We suspect that this might be because older adults were barely acknowledged as competent especially under COVID-19 circumstances (Fraser et al., 2020; Shahid et al., 2020), while our materials were also about patients in the pandemic. Therefore, it may also be more convenient for younger participants to describe a warm rather than a competent older adult under such context. Nevertheless, as the stereotypes of warmth and competence are somehow interrelated, a more integrated view of stereotypes should also be considered in the future.

### 4.3. Practical Implications

Our new interventions provided new insights into the practical issue of how to reduce hostile ageism. For instance, old photographs or sundries may help portray a more detailed and vivid image of a rejuvenated older adult. Older adults could also be invited to take part in this “Imagine that they were young” by providing their own real-life stories or autobiographies which could hugely contribute to facilitating intergenerational understanding. For example, Fruhauf et al. (2020) invited Israeli younger adults to visit a museum exhibition named “Dialogue with Time” about how older adults lived an active life and the study successfully reduced their ageism attitudes. However, we should also take precautions against “young worship” when we suggest they imagine rejuvenated older adults. The purpose of “Imagine that they were young” was never a rejection or denial of age and aging, but always the emphasis on how older and younger adults could be perceived as continuity in our society, and what we could do for intergenerational solidarity.

It still deserved attention on how older adults would be portrayed negatively in COVID-19 (Jen et al., 2021; Ng et al., 2021), due to which we urged for more discourse to empower older adults through mass media (Lytle and Levy, 2019; Levy et al., 2021). Considering this online pathway, we could further try a combination of “Imagine that they were young” and online social media (just like what we did in the present study) to encourage more younger adults to join us and make changes.

### 4.4. Limitations and Future Directions

Several limitations should also be acknowledged. First, due to the COVID-19 restrictions, we recruited participants through the online platform, which may hinder our interventions to some degree. Although the online environment allowed looser social desirability, unfortunately, we found one participant plagiarized an online description about aging as his own imagination. Second, we allowed free imaginations for both interventions but we did not code or interpret them in-depth. For example, although the majority of imaginations were neutral or positive, several participants imagined rejuvenated older adults with mainly miserable experiences. Thus, such negative emotion may cover their actual feelings about older adults but only reflect how they perceived themselves. Our results remained the same after removing those negative imaginations, but this still reminded us how varied contents could probably bring diverse consequences. Third, although it is found that “Imagine that they were young” could increase participants' willingness to allocate more resources to the older patient, ageism attitudes (as measured by the intergenerational tension ageism scale) failed to emerge significant differences across three conditions in any analysis. One possibility could be potential social desirability, such that behaviors might be more implicit, while the SIC scale as an explicit measure would be prone to reach a higher level during the pandemic as a social norm in China (Xi et al., 2021). Finally, we compared our results of separated mediation models (i.e., three models with different mediators, as we reported above) with the single mediation model (i.e., one model with three mediators together in parallel). We found that the mediation effect of the stereotype of warmth disappeared in the single model, which may be due to the small sample size in our study. Nevertheless, both two interventions remained effective in reducing hostile ageism through these mediators, and the level of IOS still played a mediation role only for “Imagine that they were young” instead of “Imagine that you were old” in the single model. Therefore, larger samples in future studies would help to clarify the collaboration and interaction among these mediators.

Future studies could further explore whether and how this new intervention could affect benevolent ageism (Cary et al., 2016), which may also stem from perceiving older adults as a warmer and higher level of IOS as Cadieux et al. (2019)'s studies suggested. Moreover, in the present study, we only tested the short-term effect of such intervention in the context of COVID-19, whether it could generalize to a longer period or other situations remained unknown. Hence, to apply these new interventions into our real life, it's also necessary to test how long could such desirable consequences of “Imagine that they were young” persist. Simultaneously, we should find more convincing evidence investigating whether our intervention could still function well in diverse real-life situations such as hospitals, senior homes (Wyman et al., 2018), workplaces (Naegele et al., 2018; Stypinska and Nikander, 2018), and even our households, in which older adults probably suffered from ageism.

Despite these limitations, our newly developed intervention “Imagine that they were young” indeed provided a better way to reduce hostile ageism compared with the widely-used “Imagine that you were old” one. Beyond perceiving older adults as warmer after taking either intervention, the “Imagine that they were young” additionally motivated younger participants to include older adults into their self-group (the young) more and also resulted in lower hostile ageism. Our research shed light on how we could encourage younger adults to take a different perspective to perceive younger and older people as continuity belonging to a uniform society and, hence, avoid so-called “calculated ageism” (Barrett et al., 2021), as well as achieve our ultimate goals of intergenerational solidarity.

## Data Availability Statement

The raw data supporting the conclusions of this article will be made available by the authors, without undue reservation.

## Ethics Statement

The studies involving human participants were reviewed and approved by Institutional Review Board of School of Psychology and Cognitive Science, Peking University. Written informed consent for participation was not required for this study in accordance with the national legislation and the institutional requirements.

## Author Contributions

ZC and XZ designed research and wrote the manuscript. ZC performed research. Both authors contributed to the article and approved the submitted version.

## Funding

This study was supported by the National Natural Science Foundation of China (#31871121 to XZ).

## Conflict of Interest

The authors declare that the research was conducted in the absence of any commercial or financial relationships that could be construed as a potential conflict of interest.

## Publisher's Note

All claims expressed in this article are solely those of the authors and do not necessarily represent those of their affiliated organizations, or those of the publisher, the editors and the reviewers. Any product that may be evaluated in this article, or claim that may be made by its manufacturer, is not guaranteed or endorsed by the publisher.
