# Supplementary material for: We Were All Once Young: Reducing Hostile Ageism From Younger Adults' Perspective
Source: Front Psychol. 2022 Mar 24;13:793373. doi: 10.3389/fpsyg.2022.793373 (PMC8988281; doi:10.3389/fpsyg.2022.793373)
Supplement: Supplementary file 1 [file Data_Sheet_1.pdf]

Supplementary Material

1 PILOT STUDY TO TEST THE EFFECTIVENESS OF PRIMING ON HOSTILE AGEISM

We recruited 145 younger adults on an online platform (average age= 29.67, SD=4.88, Female = 49.0%) and ask them to read one of three passages about older adults in COVID-19. These three passages emphasized respectively the illness and deaths among older adults (threat-of-death), need and competition on scarce public health goods (finite resource across age groups), and only the fact about infections and deaths in pandemic without stressing older adults (control). Then participants were instructed to complete the same medical-funds-allocation task. Results showed that younger participants who read the 'finite resource across age groups' essay expressed more hostile ageism than threat-of-death and control group, thus we adopted this material as priming in our main study.

2 MANIPULATION CHECK FOR PRIMING IN CURRENT STUDY

A manipulation check was included to determine whether participants who read 'finite resource across age groups' passage indeed perceived resources as scarcer and exhibited higher level of intergenerational tension than those who read similar 'finite resource' passage without stressing older adults (control condition). Another 90 participants were asked to write down a brief summary about either passage and then two coders coded the answers separately (of high scorer reliability, cohen's κ = .883 for resource scarcity and .977 for intergenerational tension). Results showed that although almost all participants in experiment (41 of 42, 97.6%) and control condition (45 of 48, 93.8%) realized the medical resource was limited, participants who read 'finite resource across age group' (38 of 42, 90.5%) mentioned intergenerational tension more than the control group (1 of 48, 2.1%, $\chi^2_{(N=90,df=1)}$ = 71.6, $p < .001$).

3 RELATIVE MEDIATION ANALYSIS WITH THREE MEDIATORS IN PARALLEL

We tested a single relative mediation model with three mediators (i.e., the level of IOS, the aging stereotype of warmth, and the aging stereotype of competence) in parallel. First of all, relative total effects reached significant for variable $\boldsymbol{D}_1$ (i.e., "Imagine that they were young" condition, 95% Bootstrap CI = [4.34, 15.42], $p < .001$) and marginal significance for variable $\boldsymbol{D}_2$ (i.e., "Imagine that you were old" condition, 95% Bootstrap CI = [-0.82, 9.79], p = .097).

Omnibus test of total effect of conditions on ageism behaviors showed significant ($F_{(2,202)}$ = 6.19, p = .003, partial η^2= .058). Omnibus test of direct effects of conditions on ageism behaviors showed significant ($F_{(2,199)}$ = 3.25, p = .041, partial η^2= .03).

As for the level of IOS, only the indirect effect of variable $\boldsymbol{D}_1$ (i.e., "Imagine that they were young" condition, 95% Bootstrap CI = [0.20, 3.61]) but not $\boldsymbol{D}_2$ (i.e., "Imagine that you were old" condition, 95% Bootstrap CI = [-0.54, 2.19]) reached significant.

As for the aging stereotype of warmth, neither the indirect effect of variable $\boldsymbol{D}_1$ (i.e., "Imagine that they were young" condition, 95% Bootstrap CI = [-0.90, 1.77]) nor the indirect effect of $\boldsymbol{D}_2$ (i.e., "Imagine that you were old" condition, 95% Bootstrap CI = [-0.80, 1.71]) reached significant.

As for the aging stereotype of competence, neither the indirect effect of variable $\boldsymbol{D}_1$ (i.e., "Imagine that they were young" condition, 95% Bootstrap CI = [-0.08, 2.21]) nor the indirect effect of $\boldsymbol{D}_2$ (i.e., "Imagine that you were old" condition, 95% Bootstrap CI = [-0.15, 1.97]) reached significant.

Further analysis showed significant total indirect effects for both dummy variable $\boldsymbol{D}_1$ (i.e., “Imagine that they were young” condition, 95% Bootstrap CI = [0.96, 5.85]) and dummy variable $\boldsymbol{D}_2$ (i.e., “Imagine that you were old” condition, 95% Bootstrap CI = [0.09, 4.29]).
